# Prioritizing simple administrative measures to ensure appropriate tuberculosis infection control

**DOI:** 10.1186/1753-6561-5-S6-P41

**Published:** 2011-06-29

**Authors:** D Chemtob

**Affiliations:** 1 Stop TB Department, World Health Organization, Geneva, Switzerland

## Introduction / objectives

For reducing Tuberculosis (TB) transmission, early diagnosis and prompt treatment of TB patients should be completed by implementing TB Infection Control (TB-IC) measures. Our objective is to analyze the TB-IC Global Policy, its current implementation status and strategies to scale-up TB-IC measures at country level.

## Methods

Analysis of the TB-IC Global Policy, and description of strategies and challenges for scaling-up TB-IC measures at country level.

## Results

“WHO Policy on TB-IC in Health-Care Facilities, Congregate Settings and Households” (2009) recommended the implementation of a set of measures (administrative, personal protection, environmental). Since, WHO and other partners have been engaged in a series of actions, including Regional/National trainings of hundreds of professionals and Technical Assistance to countries. A new indicator for monitoring TB-IC and the cost analysis for a worldwide implementation have also been integrated into the "Global Plan to Stop TB 2011-2015". However, TB-IC is still in a preliminary implementation phase in most of the countries. Scaling-up should therefore prioritize the implementation of simple and economical administrative measures; e.g. identifying potentially infectious cases (triage), separating them, and assuring health care worker protection. By embedding TB-IC plans into broader ones (i.e. MDR-TB, HIV, Health System Strengthening, general IC), TB-IC measures should progressively be incorporated into national plans funded by major donors. Preliminary data on country implementation, opportunities and bottle-necks will be presented.

## Conclusion

Using first simple and economical TB-IC measures, together with embedding TB-IC within broader plans, should contribute to their step-wise implementation. This should also ultimately impact positively the country TB burden.

## Disclosure of interest

None declared.

